# The Power of mind: Blocking visual perception by hypnosis

**DOI:** 10.1038/s41598-017-05195-2

**Published:** 2017-07-07

**Authors:** B. Schmidt, H. Hecht, E. Naumann, W. H. R. Miltner

**Affiliations:** 1 Institute of Psychology, University of Jena, Jena, Germany; 2 Institute of Psychology, University of Trier, Trier, Germany

## Abstract

The present study investigated the effects of suggestion on the processing of visual stimuli. Participants counted rare visual stimuli presented on a screen, once during a hypnosis condition where they were suggested that their vision of the screen is blocked by a virtual wooden board in front of their eyes and once during a control condition without suggestion. In the hypnosis condition, counting performance was about 20% worse than in the control condition. At the same time, the P3b amplitude of the event-related brain potential was about 37% reduced. Smaller P3b amplitudes were significantly associated with deficient counting performance, and this effect was largest in participants who reported the blockade as real. In contrast, earlier brain responses (N1, P2) that reflect basic processing of the visual stimuli were not affected by the suggested blockade. We conclude that the suggestion of the blockade affects later stages of visual perception, leaving early processes intact. This illustrates the impact of suggestions and the power of mind.

## Introduction

It is an astonishing power of the human mind that a suggestion during hypnosis can have a deep impact on behavior and perception^1–4^. To date, the most impressive and best-examined example for the power of suggestion is its effect on pain. Pain can be significantly reduced by suggestions of analgesia while participants are hypnotized^5^. Another impressive example is the impact of suggestions on color vision. When susceptible participants are suggested to see grey instead of colored stimuli or vice versa while hypnotized, they see what they are suggested, mirrored by corresponding changes in visual areas of the brain^1^. In our study, we show that the suggestion of a virtual perceptual blockade can significantly block one’s visual perception and the cognitive performance associated with correct and unimpaired perception.

**Figure 1:**
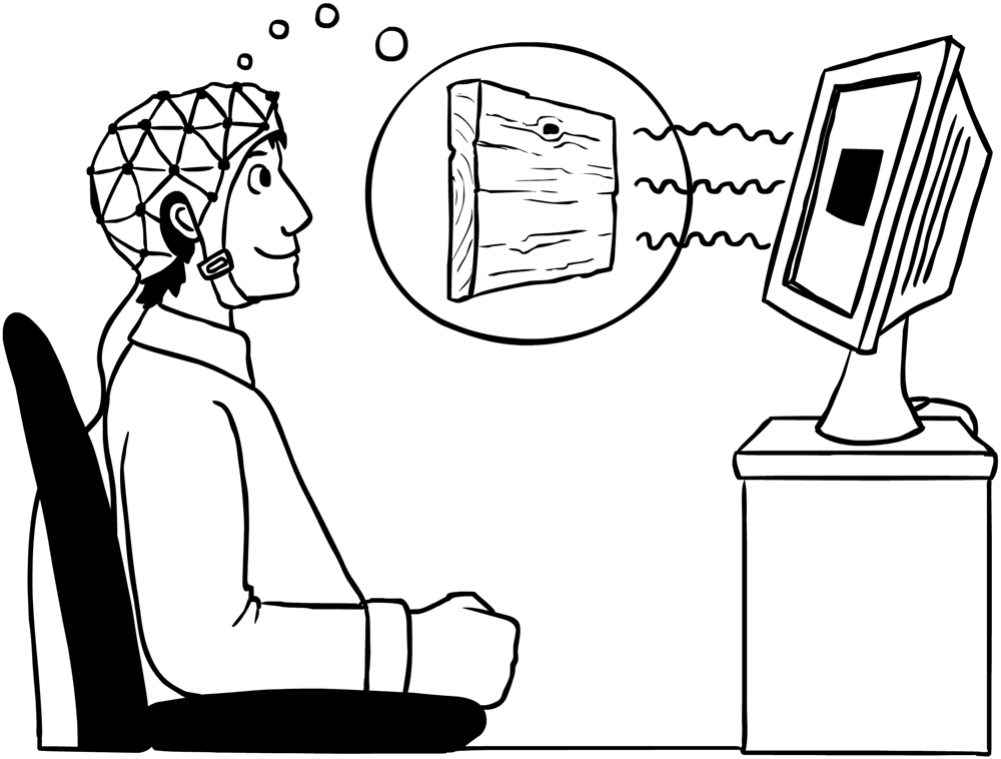
Illustration of the suggested perceptual blockade.

After the induction of hypnosis, we suggested to participants that they would see a virtual wooden board in front of their eyes blocking their view of a monitor in front of them, although being required to keep the eyes open. Figure 1 illustrates this concept. The translated text of the suggestion is provided as supplementary online material. Using a three stimulus Oddball paradigm^6^, one frequent and two rare stimuli were presented in random order on the monitor. Participants were requested to count one of the rare stimuli and leave the other stimuli unattended. Many previous studies have shown that rare to-be-counted stimuli are associated with a large late component of the event-related brain potential called P3b^6, 7^. Furthermore, it was shown repeatedly that the less probable the rare stimuli, the larger the magnitude of the P3b response^6^.

We predicted that the suggestion of a perceptual blockade should lead to deficient counting performance of the rare to-be-counted visual stimuli. Further, we hypothesized that the P3b response to these rare stimuli should be significantly reduced during the perceptual blockade compared to a control condition without blockade suggestion^8^. The amount of the P3b reduction should be associated with deficient counting performance and a more realistic experience of the blockade.

Past hypnosis research has shown that not every participant follows suggestions equally well. Therefore, we measured the individual level of suggestibility with a standardized procedure by a group test^9^ before the main experiment. We included 60 participants with different levels of suggestibility: 20 low suggestible, 20 middle suggestible and 20 high suggestible participants.

## Method

### Participants

We recruited healthy participants at the University of Jena. Participants with neurological, psychological, cardiovascular, or visual diseases were excluded from the experiment. All participants signed informed consent statements. The study was approved by the ethics committee of the Faculty of Social and Behavioral Sciences of the Friedrich Schiller University of Jena and followed the ethics declaration of Helsinki.

The assessment of suggestibility of participants was organized in groups of about 8 participants using the Harvard Group Scale of Hypnotic Susceptibility^9^. This test consists of 12 suggestions including challenge items (e.g. arm immobilization), ideomotor items (e.g. hand lowering) and cognitive items (e.g. fly hallucination)^10^. At the end of the test, participants rated whether and how well they responded on each of these 12 tasks. A total number of 0 to 3 successful tasks indicates low suggestibility, 4 to 7 moderate suggestibility and 8 to 12 tasks high suggestibility^11^.

Our sample consisted of 60 participants (30 female, 30 male) with 20 low suggestible, 20 middle suggestible and 20 high suggestible participants. Mean age of participants was 23.1 years (range 18–44 years). Participants received 6 Euros per hour for participation. On average, participants earned 24 Euros for the whole study.

### Visual Oddball task

The three types of stimuli comprising the Oddball paradigm included triangles as frequent stimuli, appearing in 80% of trials while circles and squares served as rare stimuli, each appearing in 10% of trials. Stimuli occupied a visual angle of 4.6 degrees. Each stimulus was displayed for 500 ms at the center of a monitor screen, separated by an inter-stimulus interval of 1000 to 2000 ms. Each Oddball task consisted of 500 stimuli and lasted about 10 minutes. The experiment was performed using Presentation® software (Version 17.1, Neurobehavioral Systems, Inc., Berkeley, CA, www.neurobs.com).

### EEG

EEG was recorded from 64 EEG electrodes with 21 electrodes localized according to the 10–20 system on participants’ heads. The remaining electrodes were interspaced equally between these 21 sites. The impedance of each electrode was kept below 10 kOhm. The initial sampling rate was 1000 Hz, but data were down-sampled off-line to 250 Hz. Linked mastoids (TP9 and TP10) served as reference electrodes. The EEG signal was amplified by BrainAmp amplifiers and recorded with the BrainVision Recorder software (both Brain Products, Gilching, Germany). Data analysis was realized with the EEGlab software^12^. Artifacts related to eye movements were removed with the ICA procedure of EEGlab^13^. Epochs were selected between −200 ms and 800 ms for each stimulus and condition. In order to obtain event-related brain potentials for each participant in response to stimuli, artifact-free epochs were averaged for each participant, electrode, and experimental condition. The event-related brain potentials (ERP) were baseline-corrected by the average EEG activity between −200 and 0 ms for each electrode. N1 and P2 amplitudes were assessed at electrode Fz and the P3b amplitude was evaluated at electrode Pz. Time windows for amplitudes were: 80–168 ms for the N1, 168–272 ms for the P2 and 320–472 ms for the P3b. Statistical analyses were computed with R^14^. For between-group t-tests, we used the Welch unequal variances t-test implemented in R that corrects the degrees of freedom in case of unequal variances. For ANOVA effects, we report generalized eta squared values as effect size. All reported correlations are Spearman correlations.

### Procedure

Each participant completed the hypnosis condition and the control condition in one experimental session. The order of conditions was counterbalanced across participants. An illustrative movie of the hypnosis condition in this experiment can be requested from the authors. Prior to the hypnosis condition, participants were handed a real wooden board providing a model for the imagined blockade. Hypnosis was induced by a series of verbal instructions via an in-ear microphone by a trained hypnotist from outside the experimental chamber. She mainly instructed the participants to calm down, relax, and breathe smoothly. Furthermore, suggestions also included an instruction for a motor task of the Harvard Group Scale of Hypnotic Susceptibility^9^ that served as a test whether participants were hypnotized properly.

Then, the suggestion of the perceptual blockade followed (see supplemental material). Participants were told that the board would block their perception of the screen. While they imagined the blockade, the visual Oddball paradigm was presented. Triangles were used as frequent stimuli, appearing in 80% of trials and circles and squares served as rare stimuli. The rare stimuli appeared in 10% of trials each. Thus, two kinds of rare stimuli were presented, but only one of them, the squares, had to be counted. All stimuli occupied a visual angle of 4.6 degrees and were displayed for 500 ms at the center of the screen. The interval between stimuli varied between 1000 and 2000 ms. In the control condition, participants were presented the same visual stimuli but without the suggestion of a wooden board in front of their eyes. During both conditions participants’ EEG was recorded. To make sure that participants’ eyes were open during the presentation of the visual stimuli, we used an eye tracker (SensoMotoric Instruments, Berlin) and observed a video showing participants’ eyes.

After each condition, participants were asked how many squares they counted. Based on their answers, we computed the counting performance as the percentage of correctly counted squares in each condition. At the end of the experiment, participants rated their experience of the suggested perceptual blockade on a 5 point Likert-Scale (1: “I saw all stimuli with unchanged intensity”, 5: “I did not see the stimuli any more”).

## Results

### Counting performance

**Figure 2:**
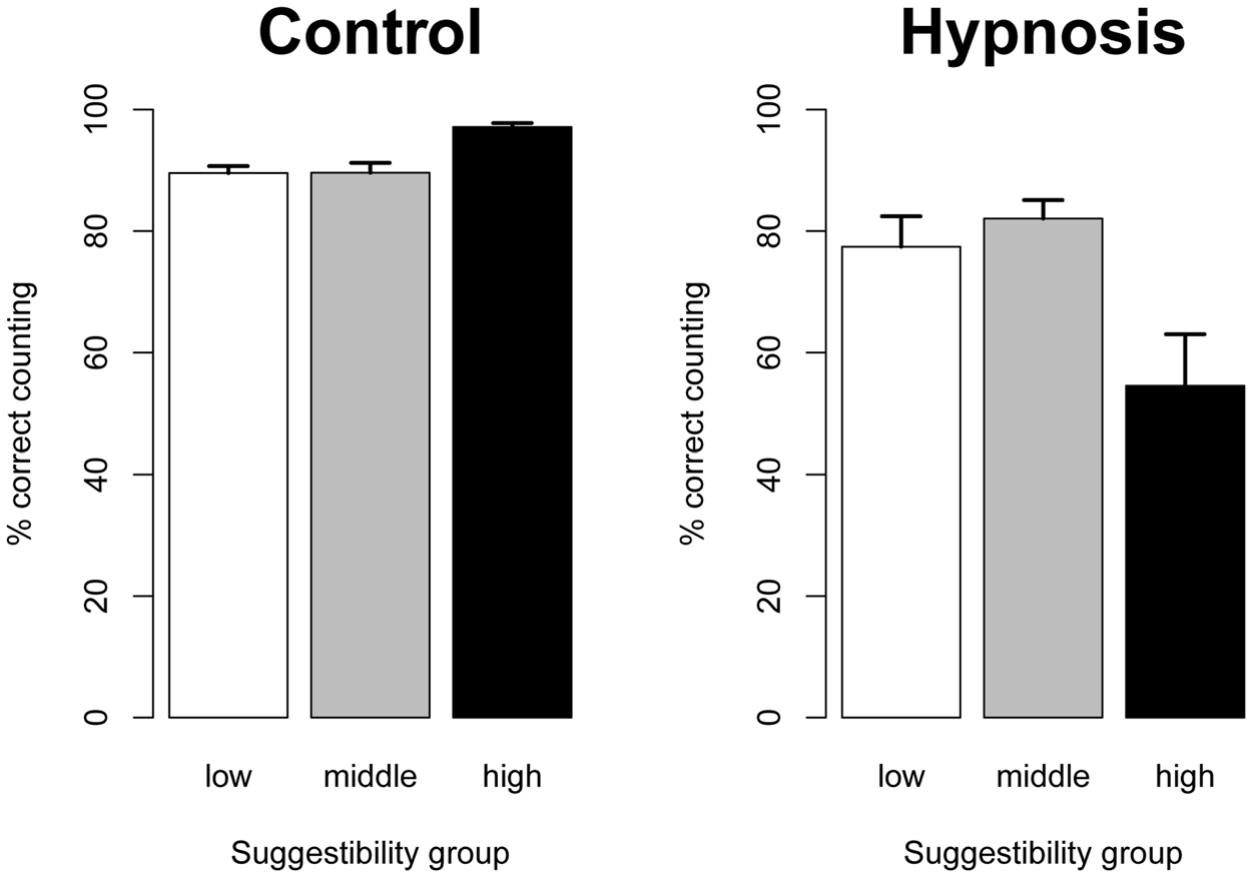
Counting performance in both conditions. Participants were very good at counting in the control condition, but significantly worse in the hypnosis condition in all suggestibility groups, especially in the high suggestible group. Error bars indicate standard errors.

To analyze counting performance, we used an ANOVA with the within-factor condition (hypnosis, control) and the between-factor suggestibility group (low, middle, high). Counting performance was significantly worse in the hypnosis condition compared to the control condition (*F*(1,57) = 33.6, *p* < 0.001, η^2^ = 0.24). This equals a mean reduction of counting performance of 20.8% in the hypnosis condition compared to the control condition. Counting performance was reduced for all participants, but the performance of high suggestible participants was the most deficient, see Fig. 2. In the hypnosis condition, they almost missed every second presented square whereas in the control condition, they were almost perfect in counting the squares, see Fig. 2. The described pattern of results was mirrored by a significant main effect of group (*F*(2,57) = 3.1, *p* = 0.05, η^2^ = 0.05) and a significant interaction of condition and group (*F*(2,57) = 9.4, *p* < 0.001, η^2^ = 0.15). As Fig. 2 suggests, participants in the high group were more accurate at counting in the control condition than participants in the middle (*t*(24.9) = 4.3, *p* < 0.001, *d* = 1.4) and low (*t*(30.0) = 5.8, *p* < 0.001, *d* = 1.8) group while there was no significant difference between middle and low group. Under hypnosis, participants in the high group made more counting errors than participants in the middle (*t*(23.8) = 3.1, *p* = 0.005, *d* = 1.0) and low (*t*(30.8) = 2.3, *p* = 0.03, *d* = 0.7) group while there was no significant difference between middle and low group. The difference in counting performance between control and hypnosis conditions was significant for the high (*t*(19) = 5.0, *p* < 0.001, *d* = 1.1), middle (*t*(19) = 2.0, *p* = 0.06, *d* = 0.4) and low group (*t*(19) = 2.3, *p* = 0.04, *d* = 0.5).

In addition, we found a significant effect of group on the subjective experience of the perceptual blockade (*F*(2,57) = 14.5, *p* < 0.001, η^2^ = 0.34) indicating that high suggestible participants had a more realistic experience of the wooden board blocking their visual perception. The association between absolute suggestibility scores and subjective experience of the perceptual blockade was also significant (*r* = 0.58, *p* < 0.001), showing the strong linearity of the effect.

A typical comment after the hypnosis condition, especially from highly suggestible participants, was “I concentrated on the wooden board and saw a board that was similar to the one you showed me in the beginning of the experiment. The board was sometimes more transparent like a cloud, so I could see the stimuli behind, sometimes more solid, so the stimuli were hidden behind it”.

### Eye blinks

We analyzed the number of eye blinks during the Oddball task via an ANOVA with the within-factor condition (hypnosis, control) and the between-factor suggestibility group (low, middle, high). Participants blinked significantly less often under hypnosis compared to the control condition: *F*(1,57) = 28.7, *p* < 0.001, η^2^ = 0.11. Please note that we handled blink related artifacts with ICA, where components representing eye movements can be selectively removed without excluding the whole trial. Therefore, the number of trials that could be evaluated for the ERP analysis did not differ significantly between conditions.

### Event-related brain potentials

For N1, P2, and P3b amplitudes of the ERP, we computed ANOVAs with the within-factor condition (hypnosis, control), the between-factor suggestibility group (low, middle, high), and the within-factor stimulus (frequent, ignored, counted).

For N1 and P2 amplitudes, only the main effect of stimulus type reached significance: N1: *F*(2,114) = 20.5, *p* < 0.001, η^2^ = 0.06; P2: *F*(2,114) = 20.7, *p* < 0.001, η^2^ = 0.05. There were no significant differences between the hypnosis and control conditions.

**Figure 3:**
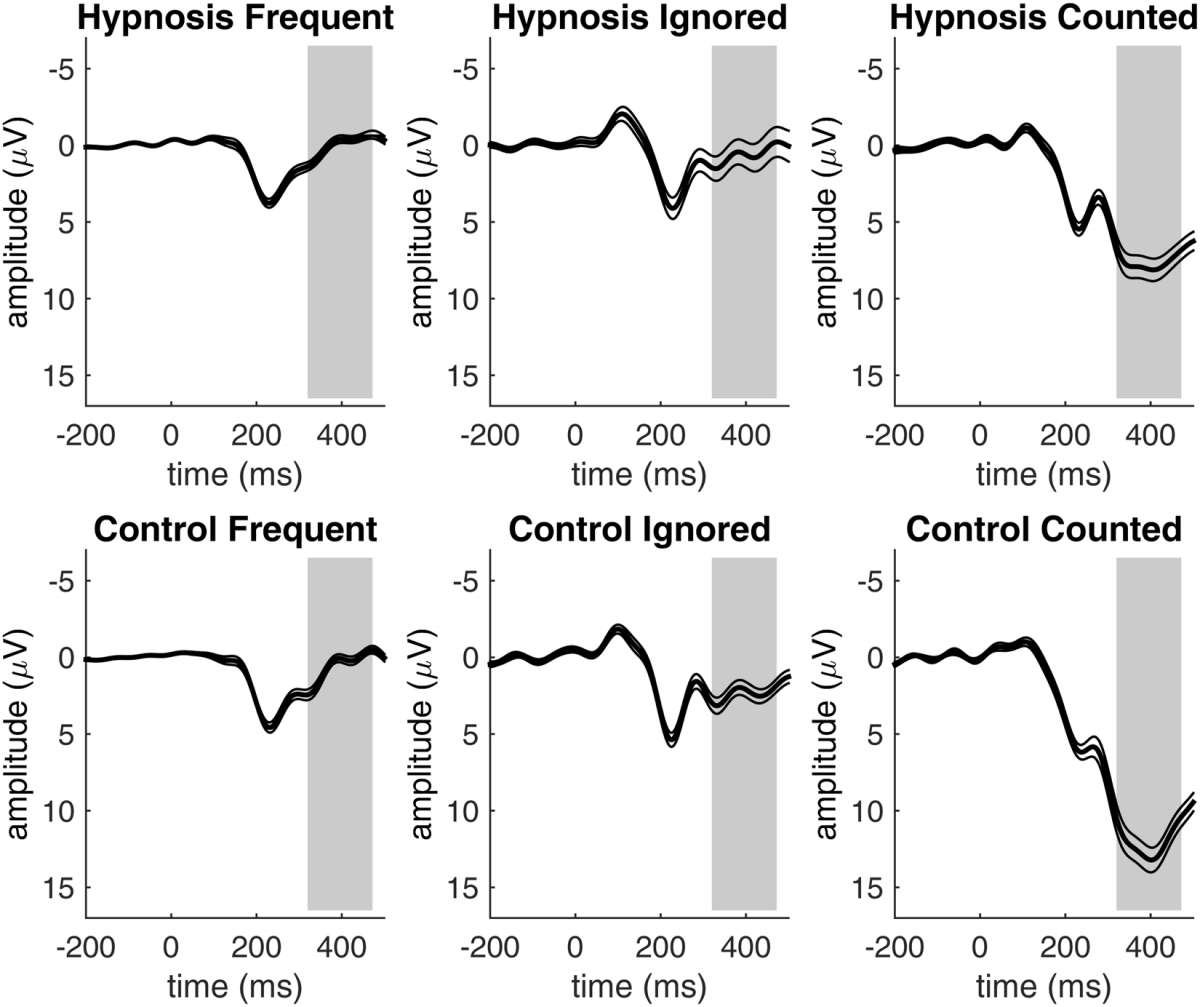
P3b amplitudes for the three stimulus types of the Oddball task in the hypnosis and control condition pooled for all participants. The Oddball effect indicated by higher P3b amplitudes for to-be-counted stimuli is significantly reduced in the hypnosis condition as compared to the control condition. Thin lines indicate standard errors, grey areas indicate the P3b time window.

For P3b amplitudes, the main effect of stimulus was significant: *F*(2,114) = 211.8, *p* < 0.001, η^2^ = 0.49. P3b amplitudes were largest for the to-be-counted stimuli, followed by the to-be-ignored rare stimuli and the to-be-ignored frequent stimuli, see Fig. 3. This is the typical oddball effect^6, 7^. P3b amplitudes were larger for the rare stimuli than for the frequent stimuli and largest for rare stimuli that had to be counted. In the hypnosis condition, the amplitudes of the P3b were significantly reduced compared to the control condition, *F*(1,57) = 22.4, *p* < 0.001, η^2^ = 0.06. This P3b magnitude reduction was observed for all visual stimuli during the hypnosis condition, but most for the target stimuli that had to be counted, mirrored by the significant interaction effect of condition and stimulus *F*(2,114) = 12.7, *p* < 0.001, η^2^ = 0.03.

**Figure 4:**
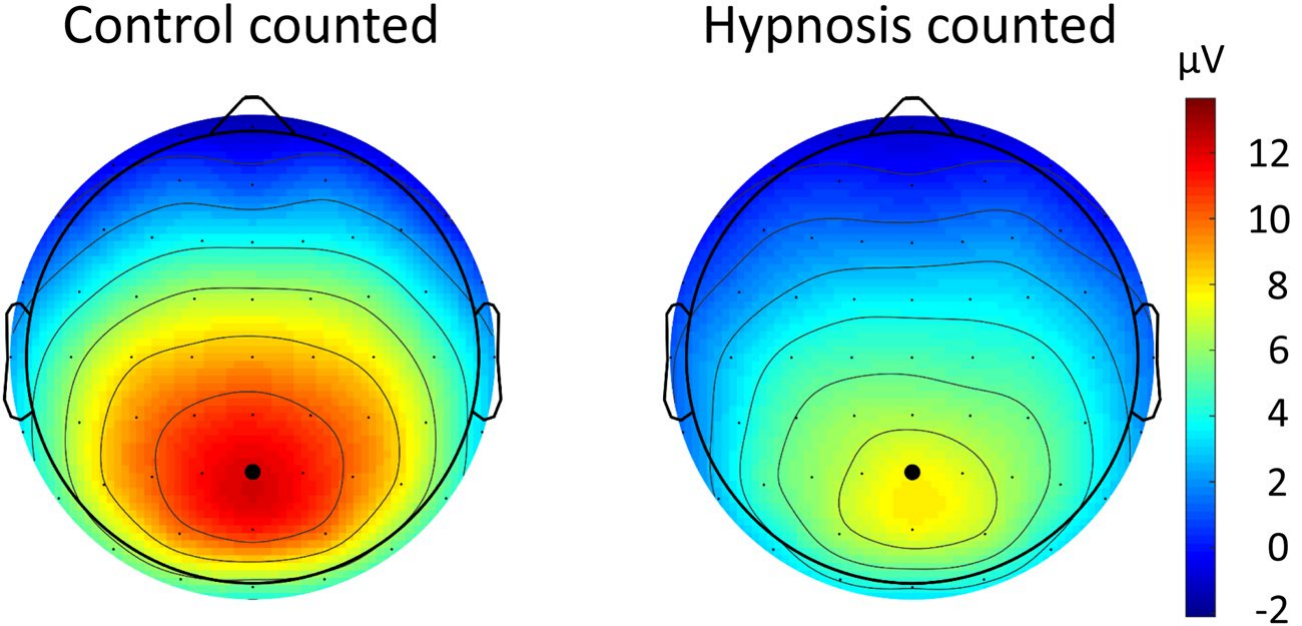
Topographical maps for the P3b response to the to-be-counted stimuli pooled for all participants, visualizing P3b magnitudes that were significantly reduced in the hypnosis condition compared to the control condition.

**Figure 5:**
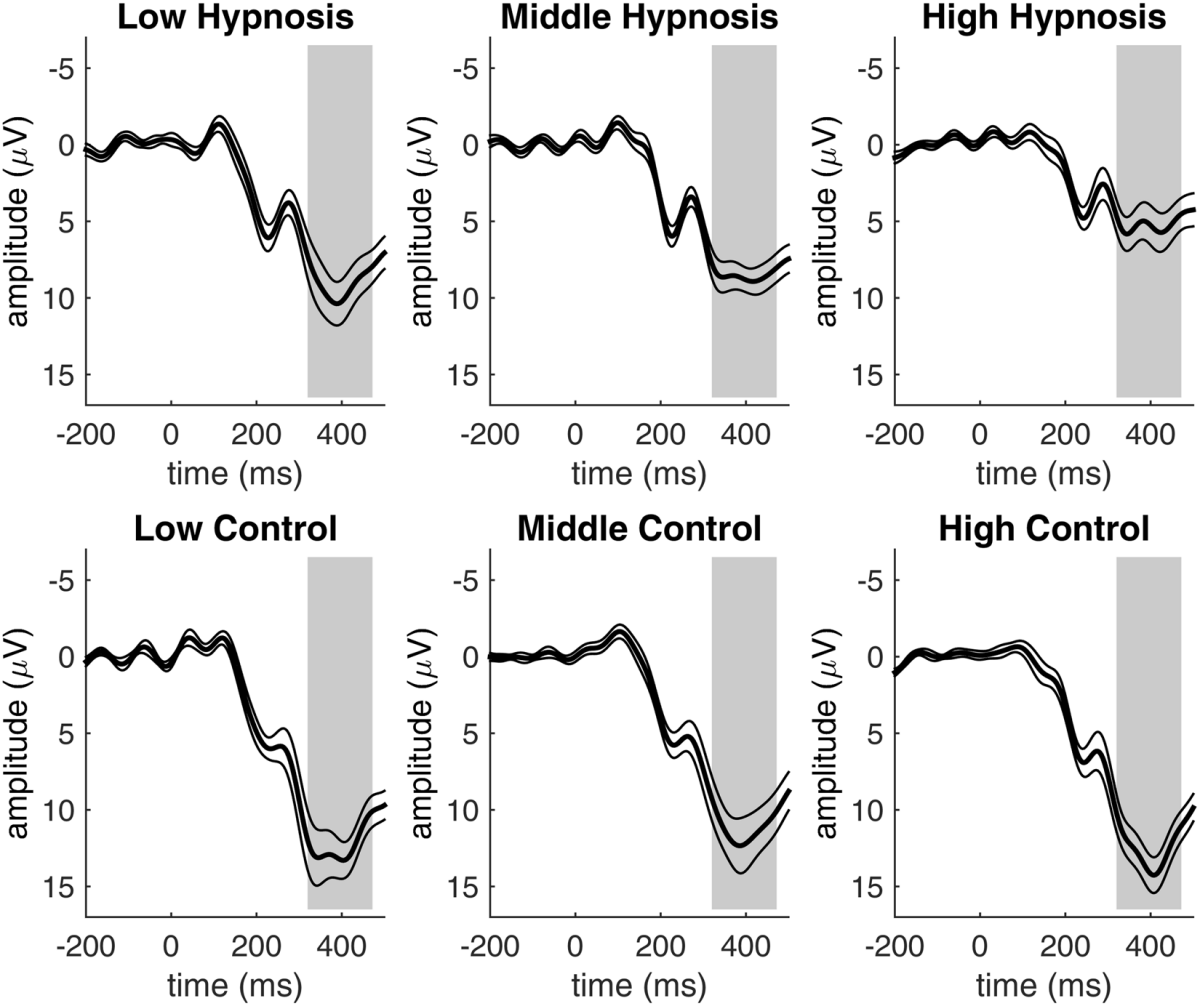
Brain responses to the to-be-counted stimuli, showing smaller P3b magnitudes during the hypnosis condition as compared to the control condition for all suggestibility groups. The grey areas indicate the P3b time window and thin lines indicate standard errors.

As we were especially interested in the P3b response to the rare to-be-counted stimuli, we conducted separate analyses only including the squares. As expected, P3b amplitudes were maximal over the parietal brain areas where P3b magnitude is commonly observed maximally. Topographical effects of the P3b reduction for the to-be-counted stimulus are displayed in Fig. 4. We also conducted an ANOVA with the factors condition and group including only the rare to-be-counted stimuli. The P3b amplitudes were significantly reduced in the hypnosis condition compared to the control condition *F*(1,57) = 44.6, *p* < 0.001, η^2^ = 0.15. The mean reduction of the P3b amplitude under hypnosis equals 37% of the P3b signal in the control condition: from 12.1 µV in the control condition to 7.7 µV in the hypnosis condition. The interaction of condition and group was also significant: *F*(2,57) = 4.9, *p* = 0.01, η^2^ = 0.04. The reduction of the P3b magnitude under hypnosis was observed in all suggestibility groups, but highly suggestible participants showed the greatest reduction, see Fig. 5.

Importantly, the amount of the P3b reduction under hypnosis was significantly associated with counting performance (*r* = 0.56, *p* < 0.001). The smaller the P3b amplitudes under hypnosis, the worse was the counting performance under hypnosis. The reduction of the P3b amplitude under hypnosis was also significantly associated with the experienced blockade (*r* = 0.54, *p* < 0.001). The more reduced the P3b amplitude under hypnosis was, the more realistic participants reported the experience of the perceptual blockade. Experience of the blockade and counting performance were also significantly correlated (*r* = 0.57, *p* < 0.001). The more realistic participants experienced the perceptual blockade, the more deficient their counting performance.

## Discussion

The present study revealed that a suggested perceptual blockade significantly impairs visual perception and the cognitive processes associated with perception. Participants had to count visual stimuli and made significantly more counting errors in the hypnosis condition when they were suggested a wooden board in front of their eyes compared to a control condition without such visual blockade suggestion. We tested three groups of participants: 20 low, 20 middle and 20 highly suggestible participants. Participants’ counting performance was about 20% worse after the blockade suggestion, with highly suggestible participants showing the biggest decrease of counting performance. In the control condition, participants in the high suggestibility group were more accurate at counting compared to participants in the middle and low suggestibility groups. We argue that highly suggestible participants generally focus better on task instructions, leading to better counting performance in the control condition and worse counting performance in the hypnosis condition. While participants counted the visual stimuli, we also measured early and late ERP responses in response to the visual stimuli. Earlier responses occurring about 100–200 ms post stimulus onset reflect basic sensory processing^15^, whereas later responses at about 300–400 ms post stimulus onset indicate mainly higher-order cognitive processing of stimuli^6^. Early brain responses were not affected by the blockade suggestion or suggestibility of participants. But the amplitude of the later P3b brain response was significantly reduced in the hypnosis condition compared to the control condition. The magnitude of the P3b amplitude reduction under hypnosis equals 37% of the P3b amplitude in the control condition. This effect was significant for all participants, but most pronounced for highly suggestible participants. So, all participants showed worse counting performance and smaller P3b amplitudes during hypnosis no matter how suggestible they were with high suggestible participants showing the biggest effects. Interestingly, we also found that eye blinks were generally reduced during the Oddball task in the hypnosis condition compared to the control condition. As it is hard to suppress eye movements voluntarily, it can be argued that this reduction of eye movements is an additional indicator of the hypnotic state^16^.

Earlier results already indicated that amplitudes of later ERP components are reduced after the suggestion of a visual blockade^8, 17, 18^. Our study shows that this reduction is significant for a big sample consisting of low, middle and highly suggestible participants and thus conceptually replicates these observations. Further, we are the first to show that the extent of the reduction is significantly associated with counting performance and the subjective experience of the perceptual blockade in the hypnosis condition. The smaller the P3b response of the brain, the more visual targets were missed and the more realistic participants perceived the perceptual blockade. The significant association of brain responses with behavior is crucial for the interpretation of these brain responses. Therefore, our results allow an important step towards understanding the mechanisms that are responsible for the effects of suggestions under hypnosis. The presence of equivalent early brain responses to the visual stimuli in both the hypnosis and the control condition indicates that participants saw the stimuli equally well in a physical sense. But later processes that are important for cognitive processes like counting visual target stimuli are affected by the suggested blockade. This is in line with the idea that suggestions under hypnosis cause a dissociation between sensory and higher perceptual processing areas in the brain. In other words, when we tell participants under hypnosis that a wooden board in front of their eyes blocks their vision, they still see the stimuli physically, but further processing of these stimuli is impaired, leading to deficient counting performance. This shows that our mind is very powerful and able to significantly modify the processing of stimuli in response to a few words of suggestion.

## Electronic supplementary material

Suggestion Text
