# Supplementary material for: The Power of mind: Blocking visual perception by hypnosis
Source: Sci Rep. 2017 Jul 7;7:4889. doi: 10.1038/s41598-017-05195-2 (PMC5501776; doi:10.1038/s41598-017-05195-2)
Supplement: Supplementary file 1 — Suggestion Text [file 41598_2017_5195_MOESM1_ESM.pdf]

Supplementary online material for the study

The power of mind: Blocking visual perception by hypnosis

by Barbara Schmidt, Holger Hecht, Ewald Naumann & Wolfgang H. R. Miltner

Translated text of the perceptual blockade suggestion

I want to try something with you now. Let’s see if you can ignore the symbol on the screen with your eyes open. (A six-sided figure is presented on the screen in front of the participant). This might sound strange as you are looking directly at the symbol on the screen. But when you are hypnotized, you can tell your brain to block things in front of your eyes so that you can’t see them anymore. Maybe you know from your daily routine that you can sometimes totally fade out things when you concentrate strongly on something different. It will be harder and harder to see the symbol. Sooner or later you will be able to fade out the symbol. Just be curious. To make that easier for you, you could imagine that I put a wooden board between your eyes and the screen. The wooden board will block your vision and keep you from seeing the screen. Just try to imagine that this wooden board moves slowly in front of your eyes so that the screen completely disappears behind it. The board is moving slowly in front of your eyes now.

You see the board in front of you, its texture and its color. Look closely! What kind of wood is it? Is it dark or light? Smooth or rough? Is there anything carved into it? The wooden board gets more and more definite in front of your eyes. You notice how hard it gets to see on the screen. You realize that you can just tell yourself to ignore the symbol. It gets harder and harder to see it as if it hides from you. It hides behind the wooden board which you see clearer and clearer now. The screen faints more and more. You notice that it gets harder and harder to see the screen.

I will start to count from one to ten and then you will see nothing on the screen anymore. One – it gets harder and harder to see the screen – two – three – the wooden board is so clear now that you can hardly see behind it – four – five – six – you are unable to look behind the board, it blocks your vision – seven – eight – nine – ten

Now you don’t see the screen anymore, the wooden board completely blocks your vision. You wonder how you made it to ignore everything else, that you are unable to see the symbol. The only thing you see is the wooden board. How fascinating it is that you can block your vision with the power of your mind! Keep on looking at the board. Look closely. You won’t see behind it until I tell you something different. Just relax. (Now the visual oddball paradigm starts)

You realize that there might be something behind the wooden board, but you don’t really care. You don’t have to care about it. Just relax. It is easy for you to ignore everything but the wooden board.
